# In Memoriam

**Published:** 1871-06

**Authors:** 


### IN MEMORIAM.

We copy the following proceedings of a meeting of the Faculty and Students of Atlanta Medical College, as a tribute of respect to our late valued friend and colleague :

ATLANTA, GEORGIA, May 10th, 1871.

Pursuant to a call, the Students and Faculty of the College met this day, in their hall, in respect to the memory of the late Professor JOHN W. JONES.

On motion of Dr. W. A. Love, Dr. W. F. Westmoreland was called to preside; and Mr. Joseph A. Meek, of Arkansas, requested to act as Secretary.

The following was submitted by Dr. Orme :

WHEREAS, In the Providence of God, our colleague and teacher, Dr. JOHN W. JONES, in the evening of life, and after a long and useful career in a profession he loved, and which he honored by his exemplary course, has been called from time; therefore,

*Resolved*, By the Faculty and Students of the Atlanta Medical College, that in the death of Prof. JOHN W. JONES, the medical profession has lost one of its ablest representatives and most zealous members ; the Faculty of the College a loved colleague, and the class an able and honored teacher.

*Resolved*, That we tender the bereaved family our deep sympathy in their great affliction ; and, that the Dean of the Faculty be directed to furnish them with a copy of these proceedings.

*Resolved*, That in respect for the memory of our late worthy colleague and teacher, all exercises of the college be suspended for to-day.

Dr. J. G. Westmoreland moved the adoption of the resolutions, and in substance, said :

*Mr. Chairman*—For twenty-five years I have known him whose death enshrouds to-day this Institution in gloom. Full fifteen years ago our acquaintance had ripened into the warmest friendship, which knew no abatement but in death. His virtues have always commanded my admiration, while with self-satisfaction I have endeavored to emulate his noble deeds of mercy and kindness.

When first we met, in 1845, a prominent and noble trait in his character was indellibly impressed upon my memory. I was then, as some of these young men, who have to-day laid aside their studies to mourn the loss of a beloved teacher, will soon be situated. My pupilage had just merged into the practical duties of life in the medical profession, and at this critical period, the frown of selfishness or jealousy, from older physicians, might have crushed the energies and blasted the prospects of my young aspirations. It may be that to the kindness and encouragement of Dr. JOHN W. JONES, our late colleague and teacher, is due what of success I have attained in the profession. Embarrassed by the responsibility of a critical case, upon the termination of which depended, perhaps, my character as a practitioner, I found in this specimen of nature's nobility, the means of success.

I have known Dr. JONES in public and in private life; professionally and in the private circle, and have always found the ruling trait in his character, and from which he seemed to derive the greatest enjoyment, that of making others happy.

Having a desire, always to please, he combined affable manners with an observance of the golden rule—"do unto others as you would have them do to you;" and although sensitive to an intended wrong, was always ready to make allowance for the frailties of mankind.

With this college he was identified at its organization, in 1855, and has always taken a lively interest in its prosperity. The loss of sight, during the war, caused him, though with much regret, to sever his connection with the Institution, on resuming exercises in 1865. Recovering from this affliction sufficiently to discharge his duties in the college, he was again elected last September, to the chair of "Practice of Medicine." He manifested great pleasure in being able to resume his duties as teacher, and less than a month since was zealous in the cause of science.

How uncertain are all things! Instead of receiving the benefits of his teaching, we are called upon, amongst the first exercises of the college, to pay the last tribute of respect to our lamented colleague and teacher.

